# In Vitro Analysis of the Accuracy of the Use of Waste Zirconia Dust Compared With Optical Scanning Spray on Implant Abutment Models in Extraoral Scanning Protocol

**DOI:** 10.7759/cureus.61633

**Published:** 2024-06-04

**Authors:** Urvi R Echhpal, Nabeel Ahmed, Ram Kiran

**Affiliations:** 1 Prosthodontics, Saveetha Dental College, Chennai, IND

**Keywords:** sustainable dentistry, zirconia waste, zirconia dust, easy scan, extraoral scanner

## Abstract

Introduction

The evolution of computer-aided design/computer-aided manufacturing (CAD/CAM) systems has heightened the significance of digital models in dentistry, particularly for fabricating prostheses like inlays, crowns, and bridges. While digital dentistry offers enhanced speed and precision, the initial investment in intraoral scanners may pose a barrier for some clinicians. Extraoral or lab scanners, however, offer a viable alternative, reducing laboratory time and providing accurate prostheses fit, though challenges such as reflective surfaces and availability of scanning sprays persist, impacting scanning quality and operator technique.

Optical scanning using laboratory scanners is a routine practice in today's age of digital dentistry. Often these require powder opacification to record fine details. There are numbered studies on the accuracy of scanning sprays.

Materials and methods

Ten casts, poured with type 4 dental stone (Elite Rock, Zhermack, Italy) with single implants, were used for the purpose of this study. Each cast was scanned by two different operators, using both mediums. It was scanned using an extraoral scanner (E4, 3Shape, Copenhagen, Denmark). Operator A used easy scan (Alphadent, Korea), followed by zirconia dust (Upcera, Guangdong, China), whereas operator B used zirconia dust first. Digital models within each group were superimposed individually to measure precision.

Results

Easy scan operator 1 and zirconia dust operator 1 differ by 0.16000 (p = 0.0802). In scenario 2, easy scan operator 2 and zirconia dust operator 2 differ by 0.21900 (p = 0.0212) . Operator type significantly affects performance, emphasizing the need to account for operator variability in relevant contexts. The trueness values obtained for zirconia dust and easy scan among both operators were statistically insignificant.

Conclusion

Zirconia dust can be reliably used for extraoral scanning of abutments in place of optical scanning sprays.

## Introduction

The importance of digital models has increased after the evolution of computer-aided design/computer-aided manufacturing (CAD/CAM) systems [1]. Manufacturing prostheses using indirect methods successfully can be an accomplishment in dental practice, especially inlays, crowns, and bridges. With the revolution of dentistry, more individuals want to incorporate digital dentistry into their routine dental practice, but not all clinicians can afford the initial investment of intraoral scanners. In order for them to incorporate the speed and precision of digital dentistry, extraoral or lab scanners commonly play a major role [2]. The use of extraoral scanning of impressions and models has allowed for shorter laboratory time, providing prosthesis with accurate fit [3]. Digitally fabricated crowns are found to have increased patient comfort and long-term low cost [4]. This hybrid technique, when used for implant level impressions, involves making a open or closed tray impression, disinfection, followed by cast pouring. After a jig trial, a suitable abutment is chosen, which is followed by scanning for designing of prosthesis. As metal is a reflective surface, it must be mattified, usually done using commercially available scanning sprays containing fine or ultrafine particles in an alcohol solvent.

Zirconia dust is a freely available material, usually disposed off as waste from milling machines. Optical scanning sprays are expensive, messy to use, and difficult to clean after scanning of models, specifically in implant abutment scans, which are used intraorally post scanning. If not cleaned correctly, they may serve as an unpolished breeding ground for microbes. Optical three-dimensional scanners detect the geometry of objects with the help of reflected light from the surface [5]. Based on this principle, the scanning quality can be influenced by reflective abilities of a surface [5,6]. The use of laboratory scanners for scanning of implant abutments is often challenging, as they are reflective and shiny , and multiple adaptive scans with the use of optical scanning sprays are required. The optical sprays used for scanning are not only expensive but also not easily available in developing nations. The use of spray differs between operators, and newer technicians and clinicians tend to heavily coat models prior to scanning which often results in extra cement gap due to increased thickness of optical spray used [7]. Improper scanning protocol of models results in misfit of restorations, eventually leading to their failure. 

Zirconia dust is a material discarded in abundance, and not many have found good use for it. CAD/CAM being a subtractive technology leaves laboratories with innumerable blanks after no more files can be nested . Dental zirconia milling dust (~30%) and block residuals (~80%) as wastes have been reported that incur significant economic and environmental losses [8].

The aim of this study was to check the accuracy of using zirconia dust as compared to commercially available easy scan optical scanning spray. The null hypothesis states that there is no difference in the accuracy of scans obtained using easy scan and zirconia dust. 

## Materials and methods

Study setting

This study was conducted at a laboratory at a dental college in Chennai. Research clearance for this study was obtained from the scientific review board of the university (SRB/SDC/PROSTHO-2106/24/004). 

Estimation of sample size 

The sample size of this in vitro study was calculated using G*Power software version 3.1.2. (Heinrich Heine University Düsseldorf, Düsseldorf, Germany) based on a study by Kang et al. with p < 0.50, significance of 5%, and power of the study of 0.95 [9]. The sample size obtained was 10. In order to eliminate bias from operator handling, each model was scanned by two operators separately. 

Obtaining the test groups

**Figure 1:**
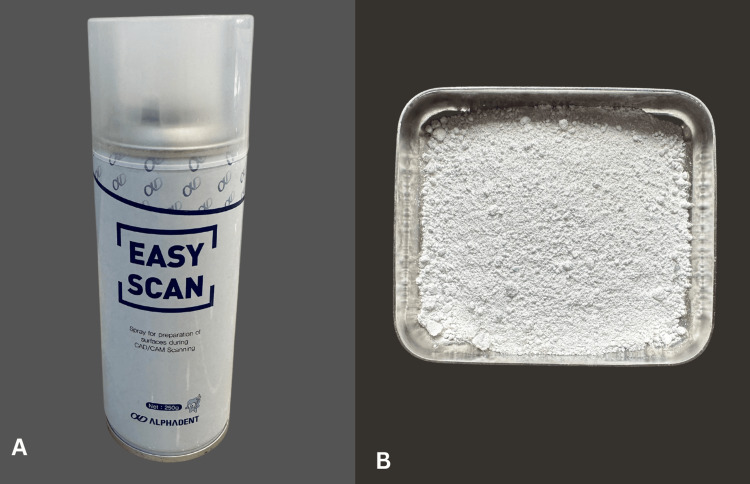
Scanning armamentarium. (A) Easy scan (Alphadent, Korea), (B) zirconia dust collected in a sterile bowl

The optical scanning spray easy scan (Alphadent^TM^, Germany) was purchased at a dental equipment store in Chennai. Zirconia dust was collected using a sterile bowl in a dry milling machine (IMES ICORE, CORiTEC^R^ 350i series, Hesse, Germany ) during milling of zirconia blanks (Upcera, Shenzen) (Figure 1).

Procedure

**Figure 2:**
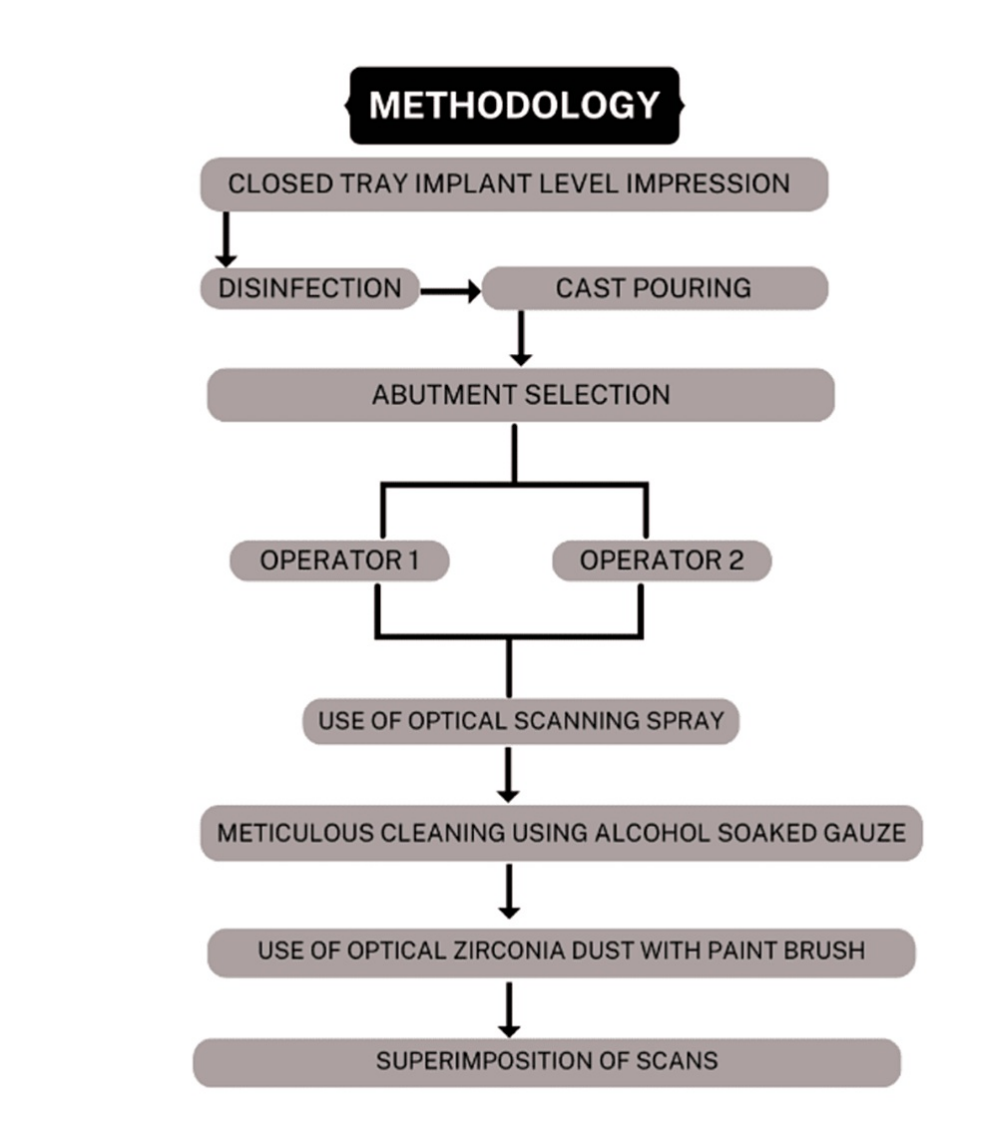
Methodology of the study

Conventional closed tray implant level impressions were taken for individuals requiring restoration of single implants using Putty and Light Body addition silicone (Elite HD+, Zhermack, Italy), which were disinfected for 10 minutes and stored for 30 minutes. Type IV dental stone (Elite Rock, Zhermack, Italy) was used for cast pouring. Post retrieval, a suitable abutment was selected and screwed into the model (Figure 2).

**Figure 3:**
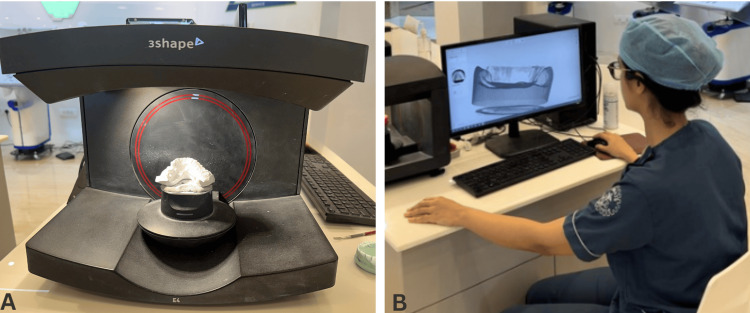
Scanning setup. (A) E4 laboratory scanner (3Shape, Denmark), (B) scanning of specimen

Optical scanning spray (easy scan) was used as per the manufacturer's instructions. The can was shaken for 30 seconds, following which the abutment was sprayed from a distance of 15-20 cm away. The spray was allowed to dry for 15 seconds, following which the scan was carried out using the E4 laboratory scanner (Figure 3). The abutment was then removed from the cast and cleaned using alcohol-soaked gauze, until no remnants were seen.

The abutment was then screwed onto the cast, and a number 8 round paint brush (Faber-Castell tri-grip) was used for the application of zirconia dust onto the abutment, and excess powder was brushed off, until no grains of dust were seen on the abutment. The cast was scanned, and the standard tessellation file was saved. 

Each cast was scanned by two operators, using both scanning mediums, to limit bias caused by methodology of the operator. The scans were superimposed using Geomagic Control X. The process involved the superimposition data from the first model as a reference and the second model as measured data. The initial overlay was initiated by activating the Initial Alignment function, followed by refining it using the Best Fit Alignment function. 

Distances between corresponding points on both models' surfaces were then computed, which could have either positive or negative values [10]. The 3D compare feature was used to demonstrate color-coded maps illustrating the superimposition variances were obtained [11]. Optimal value ranges of (+20, −20 μm) and extreme ranges of (+120, −120 μm) were selected. Subsequently, a report detailing the color differences in the superimposed models was generated as a PDF file. Deviations in the alignment (gap distances) across the entire dental arch were exported as CSV files, which were further processed in an Excel sheet. The highest and lowest 5% of deviation values for each overlay were identified and removed. An average of the absolute values of the remaining 90% of deviations was then calculated to represent the average deviation for each overlay, and the root mean square (RMS) values were obtained. To check for the trueness of each material, the scans of operators a and b were superimposed to check for the repeatability of material irrespective of operator.

**Figure 4:**
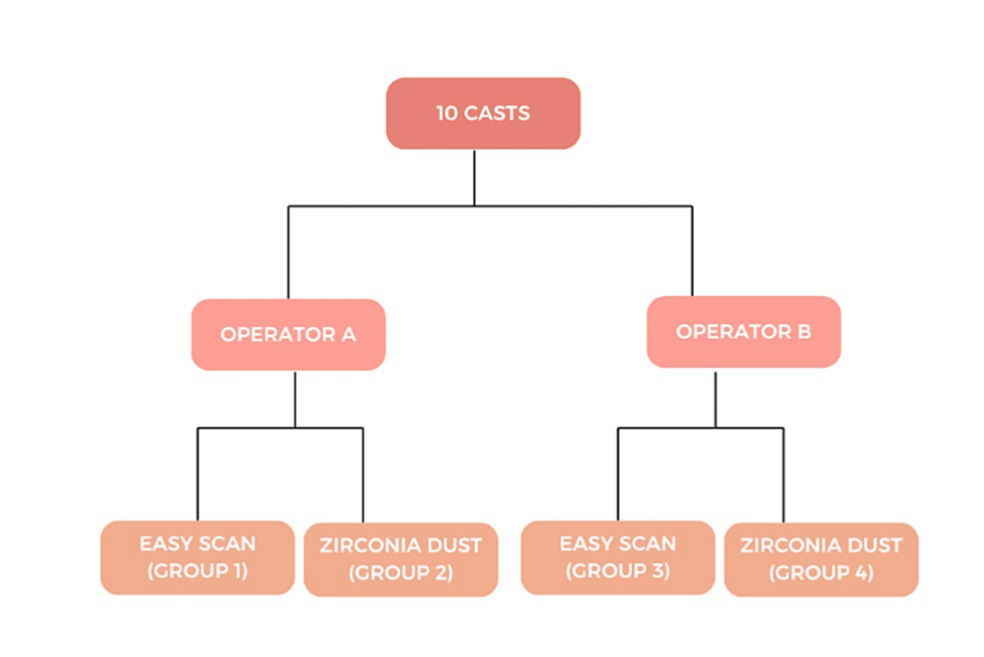
Superimpositions performed for trueness and precision

The superimpositions conducted were group 1 and group 3, to assess the trueness of easy scan; group 2 and group 4, to assess the trueness of zirconia powder; group 1 and group 2, to assess the precision of zirconia dust using optical spray as control by operator 1; and group 3 and group 4 to assess the difference in two mediums by operator 2 (Figure 4).

Statistical analysis

The RMS values for operator a and b superimpositions were tabulated using Google Sheets. The RMS value for superimposition of casts 1 to 10 were tabulated separately for operators A and B. This data was transferred to IBM SPSS Statistics for Windows, Version 26 (Released 2019; IBM Corp., Armonk, New York, United States) to run statistical analysis at a significance level of α = 0.05.

The Shapiro-Wilk tests were employed to evaluate whether the sample followed a normal distribution and exhibited homogeneity. T test was conducted in order to check for trueness. Trueness across all study groups was evaluated using the one-way analysis of variance (ANOVA) test. To demonstrate differences in groups, post hoc test was conducted.

## Results

Trueness

To check for the trueness of each material, the scans of operators a and b were superimposed to check for the repeatability of material irrespective of operator (Figure 4). The superimposition of group 1 and group 3 reveals no significant difference (p = 0.2942) between easy scan operator 1 and easy scan operator 2, with a confidence interval of 0.1862-0.2942. Similarly, the superimposition of group 2 and group 4 revealed no significant difference (p = 0.911) between both operators with a confidence interval of 0.1812-0.2992 (Table 1).

**Table 1:** Mean and standard deviation of RMS values after superimposition of all groups RMS: Root mean square

GROUP	COMPARISON GROUP	MEAN DIFFERENCE	STD. ERROR	SIG	95% CONFIDENCE INTERVAL	
					LOWER BOUND	UPPER BOUND
Easy scan operator 1	Zirconia dust operator 1	0.16000	0.8917	0.293	0.0802	0.4002
	Easy scan operator 2	0.05400	0.8917	0.930	0.1862	0.2942
	Zirconia dust operator 2	0.21900	0.8917	0.085	0.0212	0.4592
Zirconia dust operator 1	Easy scan operator 1	0.16000	0.8917	0.293	0.4002	0.0802
	Easy scan operator 2	0.10600	0.8917	0.638	0.3462	0.1342
	Zirconia dust operator 2	0.05900	0.8917	0.911	0.1812	0.2992
Easy scan operator 2	Easy scan operator 1	0.05400	0.8917	0.930	0.2942	0.1862
	Zirconia dust operator 1	0.10600	0.8917	0.638	0.1342	0.3462
	Zirconia dust operator 2	0.16500	0.8917	0.267	0.0752	0.4052

Precision of scans

**Figure 5:**
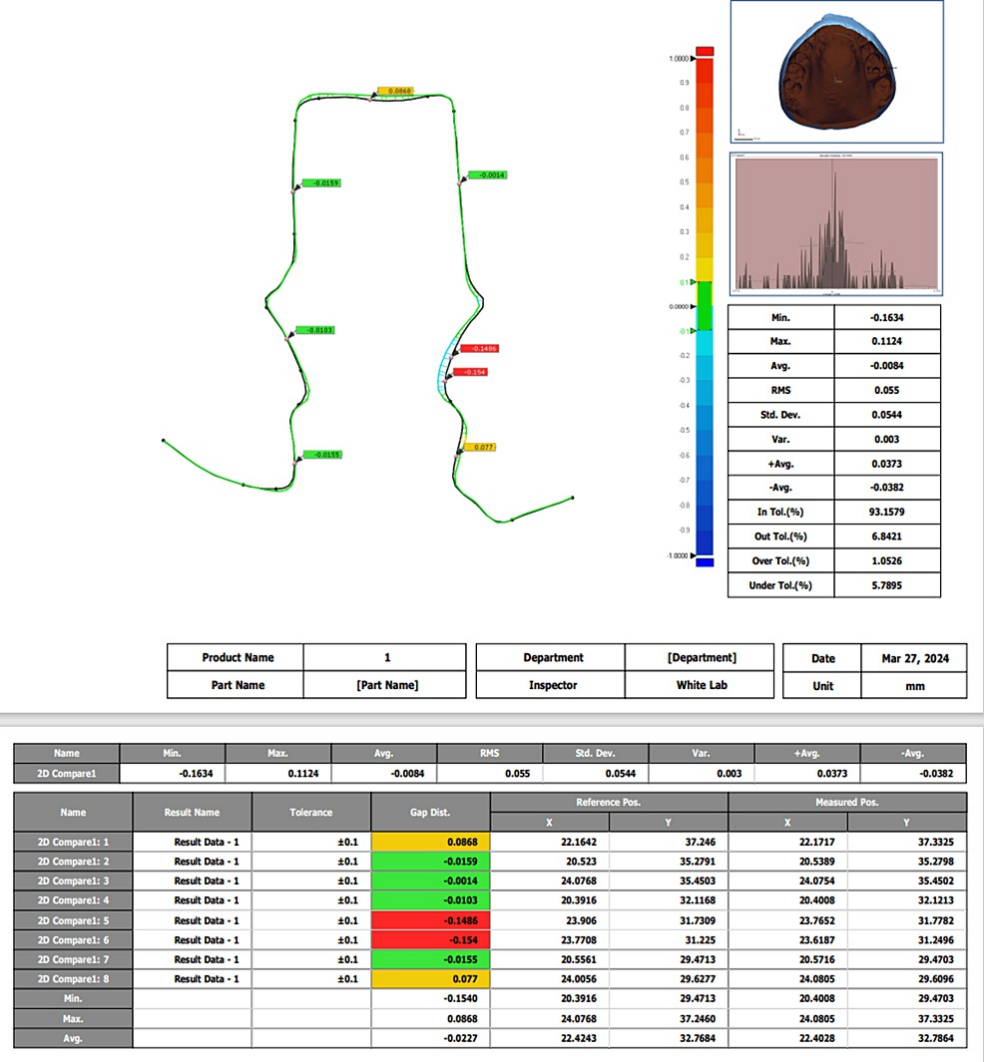
Superimposition of cast scanned using easy scan by operator a and operator b

**Figure 6:**
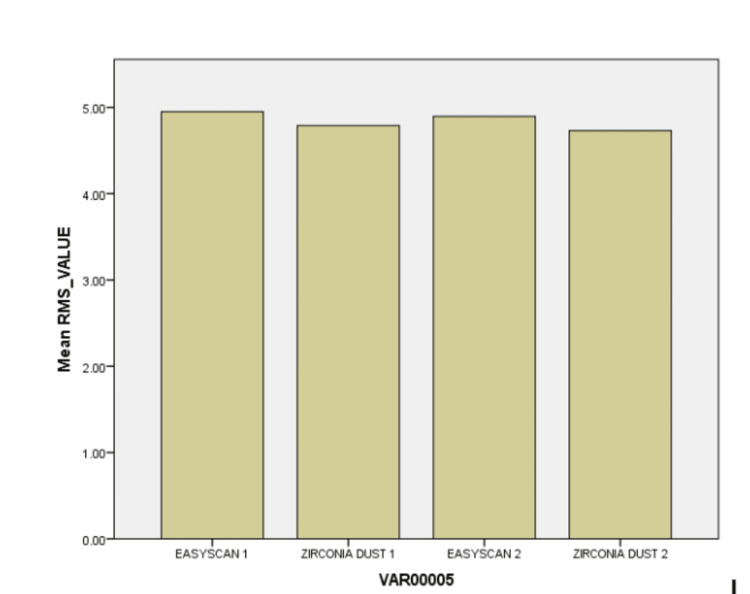
Bar graph demonstrating the trueness and precision observed of superimposed scans

In scenario 1, a statistically significant mean difference of 0.16000 (p = 0.0802) is observed between easy scan operator 1 and zirconia dust operator 1, with a 95% confidence interval ranging from 0.0802 to 0.4002. Similarly, in scenario 2, a significant mean difference of 0.21900 (p = 0.0212) exists between easy scan operator 2 and zirconia dust operator 2, supported by a confidence interval of 0.0212 to 0.4592 (Figures 5-6). These findings underscore the influence of operator type on performance across distinct scenarios, advocating for nuanced consideration of operator variability in relevant contexts.

Need for adaptive scanning

The operators demonstrated the need for adaptive scanning, only in casts wherein the level of the analog was subcrestal or deeply placed as compared to adjacent teeth, hence increasing the chance of shadow on the implant abutment. Yet, no need for adaptive scanning was reported in favorable abutment positions using both materials.

## Discussion

The analysis comparing easy scan and zirconia dust among operators in different situations reveals interesting differences. While comparing superimpositions conducted by a single operator, there was a difference between easy scan operator 1 and zirconia dust operator 1 but stands statistically insignificant (p = 0.0802). The superimpositions by operator 2 showed a more significant difference between easy scan operator 2 and zirconia dust operator 2 (p = 0.0212). However, there's no clear difference between different Easy Scan among both operators (p = 0.2942). These findings highlight how the type of operator can affect performance but, in the current study, stands statistically insignificant.

In the field of dentistry, spray techniques are employed to apply substances that reduce reflection onto dental surfaces, such as when creating optical impressions for dental repairs [12]. However, these spray methods can result in both dentists and patients inhaling fine and ultrafine particles. It's believed that inhaling these particles could have negative effects on health [13].

This study demonstrates an accurate, simple, and economical method of extraoral scanning of implant abutments. It provides a solid reference of interoperator use on a common scanner, for both trueness and precision. This study may be a clear demonstration of the ease of lab scanning, saving both time and money. Despite having two operators, a high level of trueness was obtained on the superimposition of scans from both.

Complete digital workflow relies on the availability of scan bodies and presence of a scanner which are both high investments. In developing nations and dental laboratories in smaller towns, the use of freely available materials will not only help us reuse waste but also reliably provide quality dental care in smaller setups.

In a study conducted by Oh et al., various scanning mediums were investigated to assess their accuracy and scanning time [14]. The study revealed that liquid-type scanning materials exhibited higher accuracy compared to powder-type ones when using four different intraoral scanners. This superiority was attributed to the potential influence of saliva contamination. Additionally, optical scanning sprays, when applied to different materials, proved easier to clean with a cloth [14]. However, when used on milled surfaces, they tended to roughen, making them more challenging to clean. A study reported the utilization of an automated machine for scanning, employing an electrostatic powder. Despite its elaborate and costly nature, this method yielded reliable results [15]. In an in vitro study conducted by using different sprays, coating homogeneity was significantly higher for the experienced group than for the inexperienced group. Coatings were significantly thinner for the experienced group (43.1 ± 14.09 μm) than for the inexperienced group (70.19 ± 31.26 μm) for all crown areas (p = .007) [7].

According to this study, the use of zirconia dust can be used reliably for implant abutment scanning. Implant dentistry is becoming increasingly popular, but not all clinics and setups are equipped with scan bodies and fully digital protocols. Hybrid protocols involving conventional impressions followed by digital fabrication of prosthesis are popularly followed. A study by Burde et al. demonstrating the difference in Helling 3D Laser Scanning Anti-Glare (Laser Design Inc) and Digiscan-Spray (YETI Dental produkte) showed a higher coating thickness for Digiscan-Spray yet concluded that both sprays were reliable for scanning [16].

Acknowledging the limitations of the current study, it is important to acknowledge the use of a single extraoral scanner, as every commercially available scanner would have a deviation in scans produced. Each operator would follow slight variations in their scanning methodology; therefore, multiple operators for the same specimens would demonstrate higher variations, which can significantly alter results obtained. 

## Conclusions

Hybrid techniques of digital and conventional dentistry in developing nations require economically scaling our dental practices and laboratories. This in vitro study demonstrates that zirconia dust can provide an equivalent scan to a commercially available optical scanning spray. The use of zirconia dust was found to be easy and hassle-free due to its ability to clean after scanning the models, as abutments once scanned are then transferred intraorally for the delivery of prosthesis. Zirconia dust which is freely available in all dental laboratories can be productively utilized and contributed in a small manner to minimize waste produced by the dental fraternity.
